# Supplementary material for: Phytoplankton responses to changing temperature and nutrient availability are consistent across the tropical and subtropical Atlantic
Source: Commun Biol. 2022 Sep 29;5:1035. doi: 10.1038/s42003-022-03971-z (PMC9522883; doi:10.1038/s42003-022-03971-z)
Supplement: Supplementary file 2 — Supplementary Material [file 42003_2022_3971_MOESM2_ESM.pdf]

Supplementary Material

Phytoplankton responses to changing temperature and nutrient availability are consistent across the tropical and subtropical Atlantic

Cristina Fernández-González[1-2], Glen A. Tarran[3], Nina Schuback[4], E. Malcolm S.Woodward[3], Javier Arístegui[5] and Emilio Marañón[1-2]

[1]Departamento de Ecoloxía e Bioloxía Animal, Universidade de Vigo, Vigo, Spain

[2]Centro de Investigacións Mariñas, Universidade de Vigo, Vigo, Spain

[3]Plymouth Marine Laboratory, Plymouth, United Kingdom

[4]Swiss Polar Institute, Sion, Switzerland

[5]Instituto de Oceanografía y Cambio Global, Universidad de Las Palmas de Gran Canaria, Las Palmas de Gran Canaria, Spain.

This supplementary material includes:

Supplementary Figures 1 to 5

Supplementary Tables 1 to 7

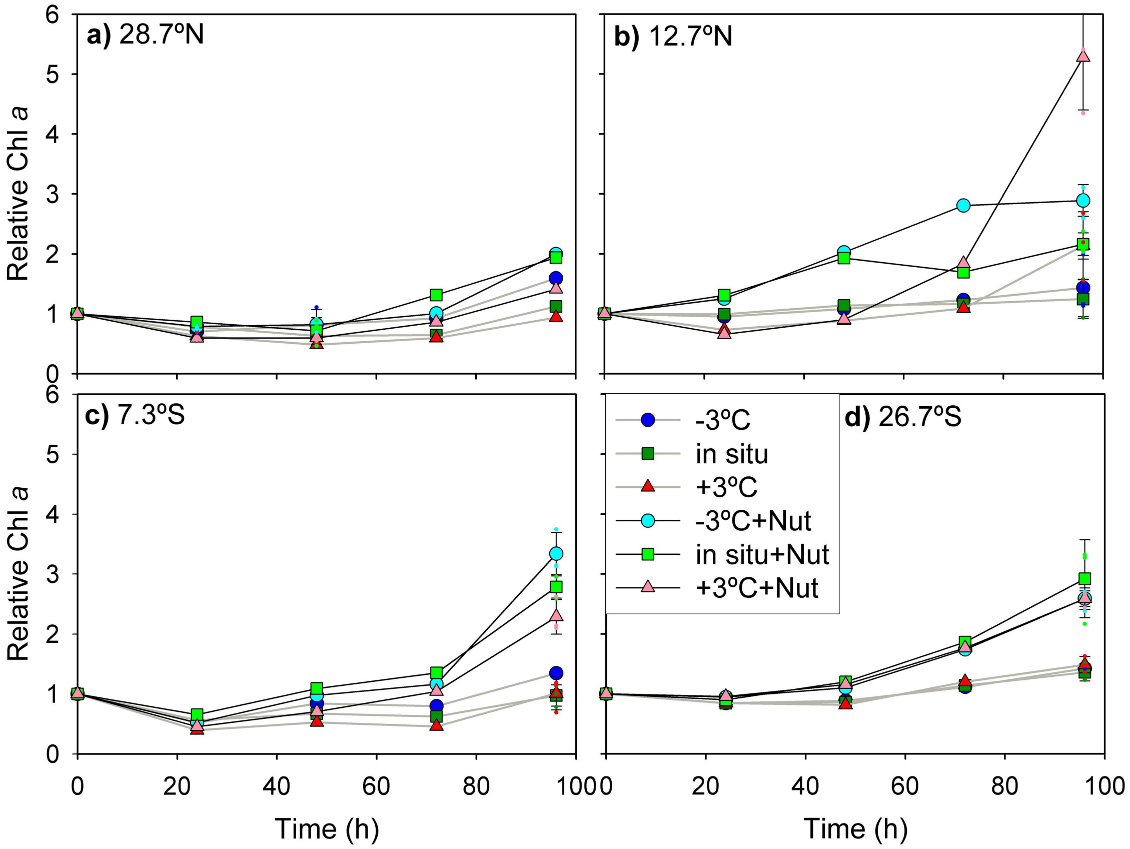

Supplementary Figure 1. Temporal evolution of fluorescence-derived chlorophyll *a* concentration during the four experiments. Data were standardized by dividing by the initial value (i.e. a t = 0 h). Symbols indicate each of the 6 different treatments, with black and grey lines corresponding to treatments with and without nutrient addition, respectively. Symbols at t = 96 h indicate the mean (n = 3) and error bars represent the standard deviation. Dots indicate each of the individual replicate measurements for each treatment.

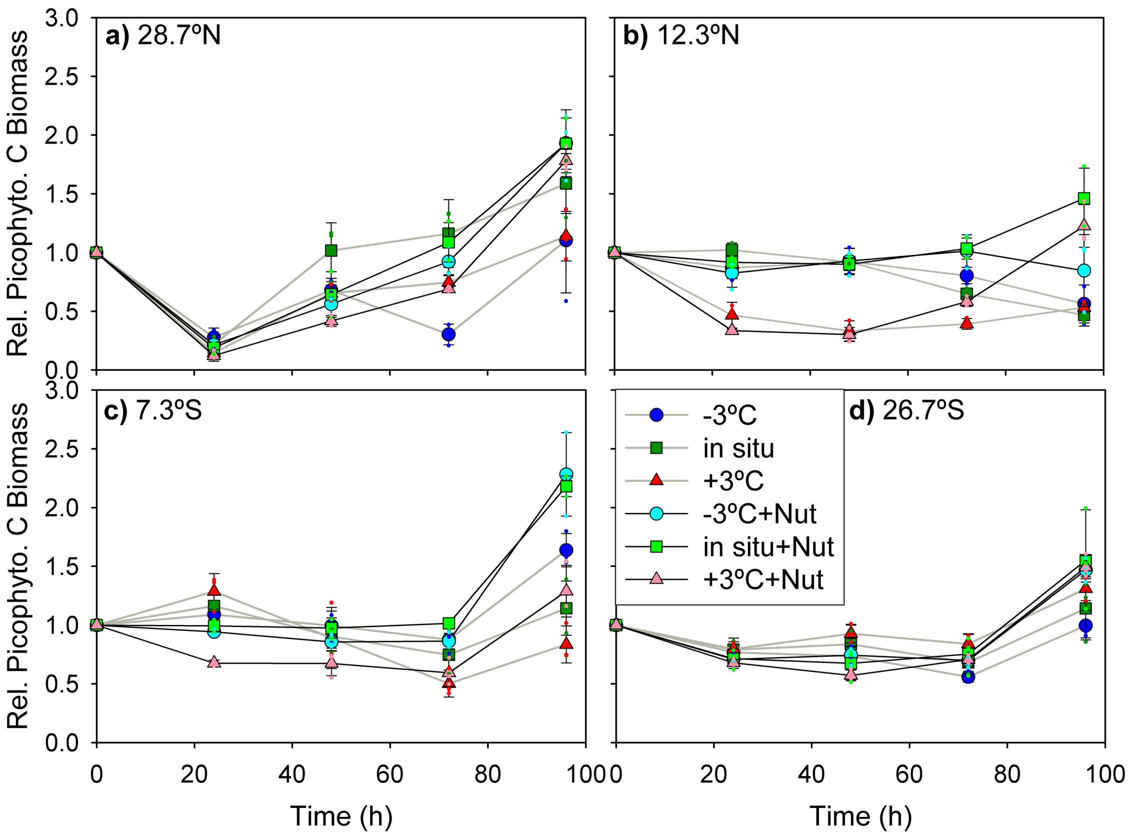

Supplementary Figure 2. Temporal evolution of relative picophytoplankton carbon biomass (i.e. standardized by the initial value) during the four experiments. Symbols represent the mean (*n* = 3) for each of the 6 different treatments, with black and grey lines corresponding to treatments with and without nutrient addition, respectively. Error bars represent the standard deviation. Dots indicate each of the individual replicate measurements for each treatment.

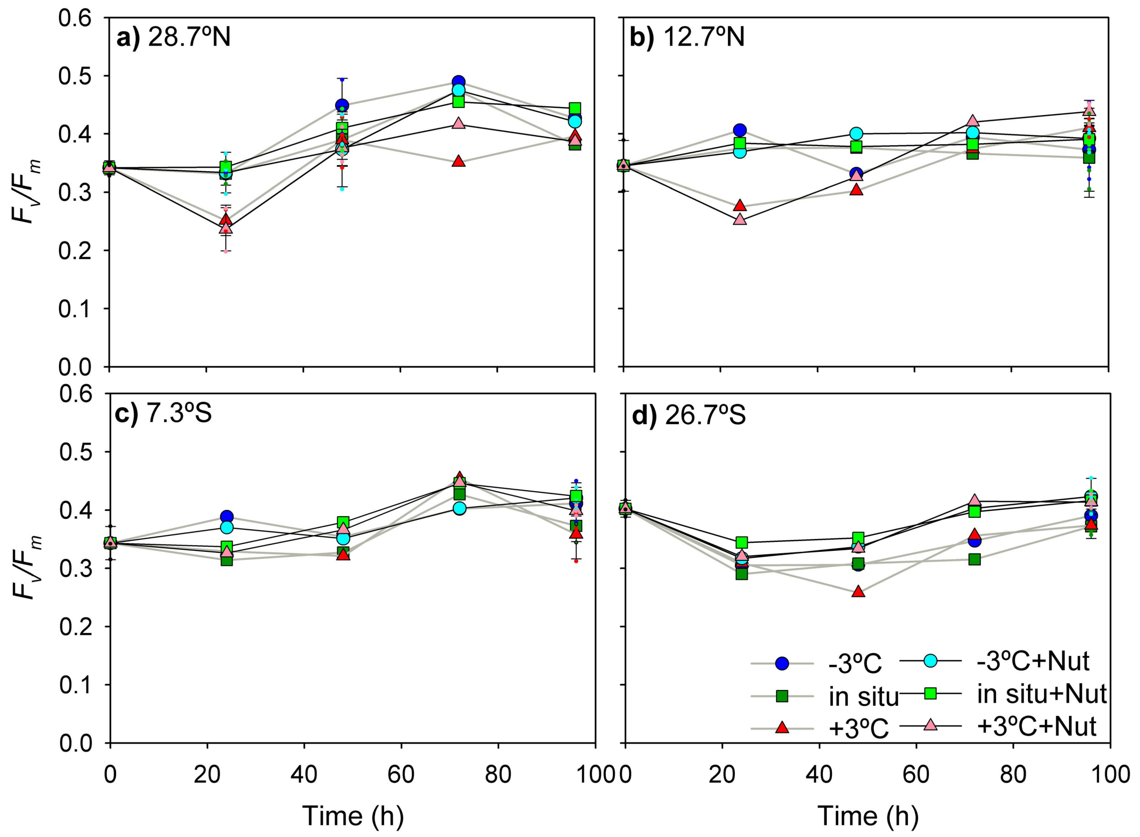

Supplementary Figure 3. Temporal evolution of F_v/F_m during the four experiments. Symbols indicate each of the 6 different treatments, with black and grey lines corresponding to treatments with and without nutrient addition, respectively. Symbols at t = 96 h indicate the mean (n = 3) and error bars represent the standard deviation. Dots indicate each of the individual replicate measurements for each treatment.

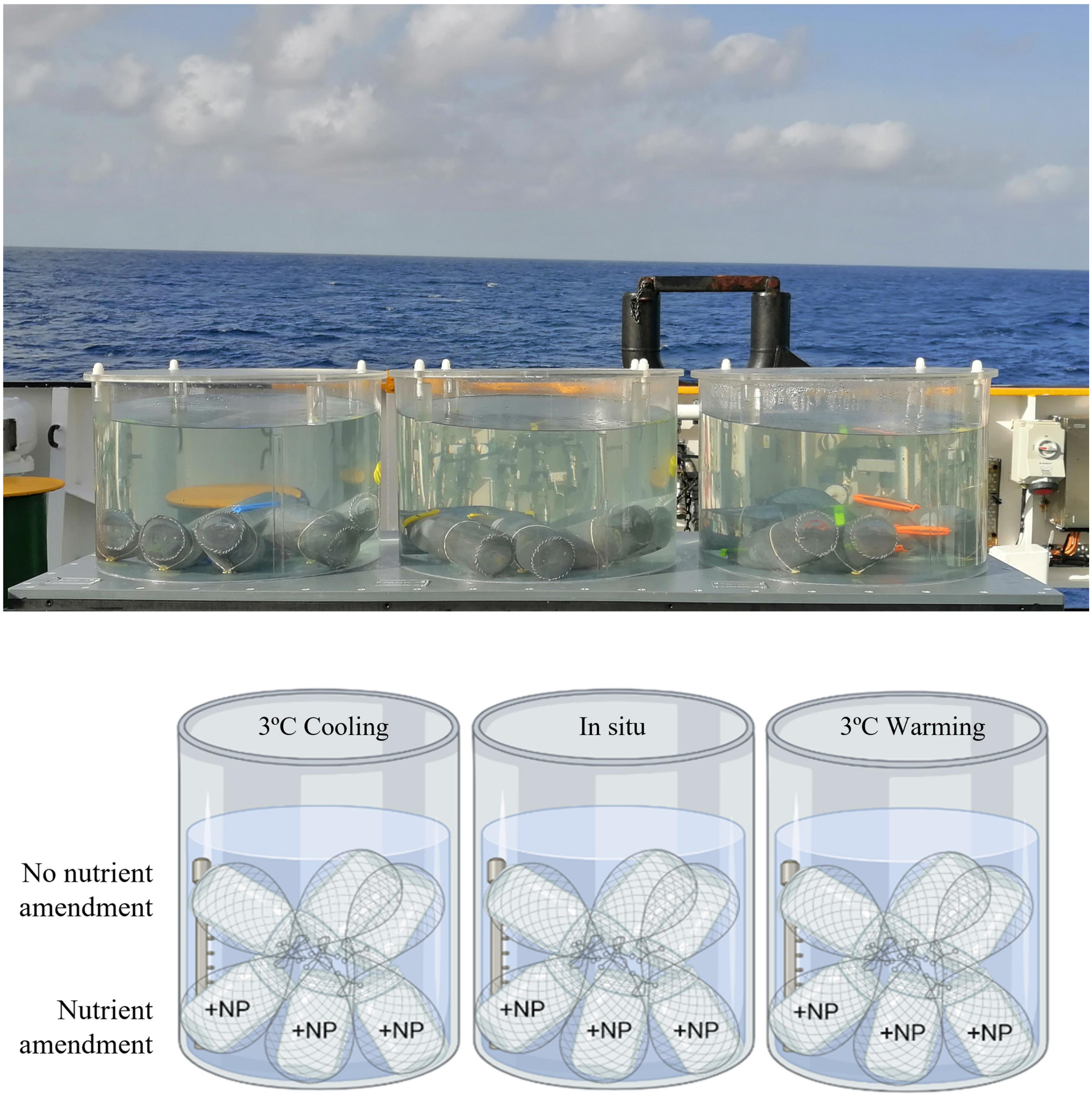

Supplementary Figure 4. Setup for each experiment conducted in the Planktotherm incubator (pictured above).

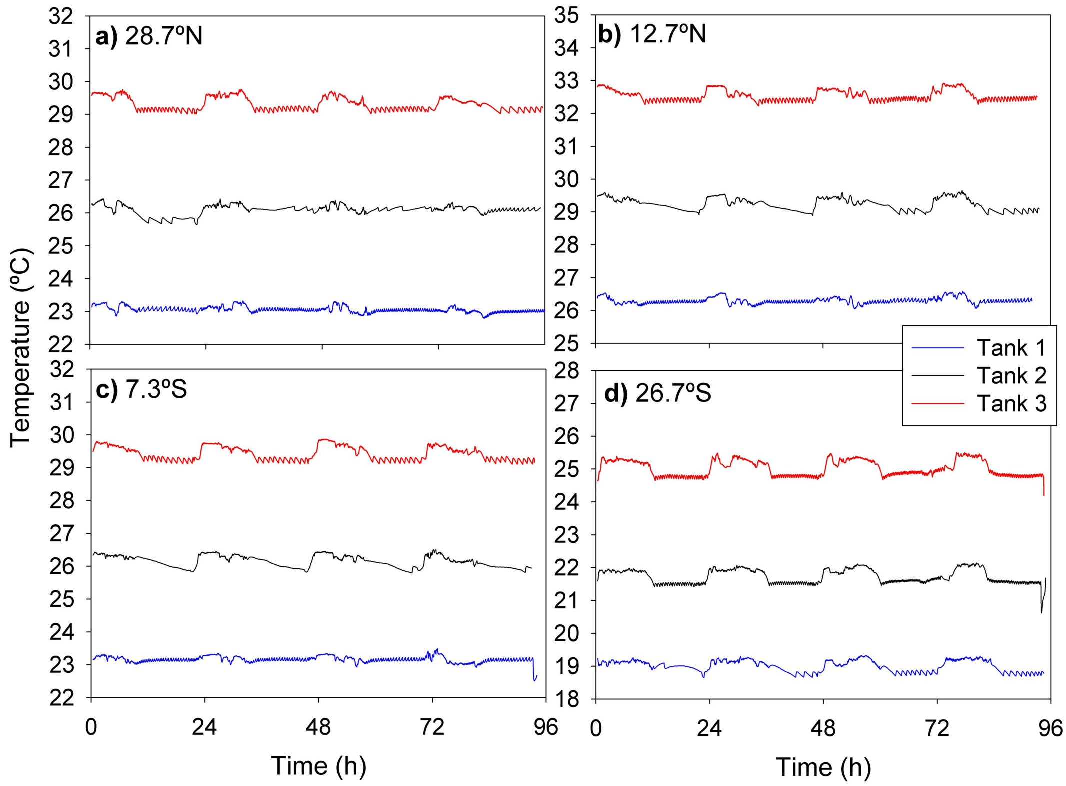

Supplementary Figure 5. Incubation temperature during the experiments. Data were recorded every 2 minutes in each tank corresponding to 3°C colder than in situ temperature (tank 1, blue), in situ (surface) temperature (tank 2, black) and 3°C warmer than in situ temperature (tank 3, red) for the experiments at **a)** 29°N, **b)** 13°N, **c)** 7°S and **d)** 27°S.

Supplementary Table 1. Analysis of differences in picophytoplankton biomass between treatments and the control. The table shows the results obtained in the one-way ANOVA test and the *p*-values for the subsequent Dunnett's post-hoc test to assess the difference in biomass between each temperature-nutrient treatment and the control. The analysis was carried out with the untransformed biomass data for each picophytoplankton group in the control and the treatments at t = 96 h on each experiment.

	ANOVA		Post hoc *p*-value				
	F(5,12)	p-value	–3°C	+3°C	–3°C +Nut	in situ +Nut	+3°C +Nut
28.7°N							
Prochlorococcus	3.67	0.03	0.34	0.74	0.03	0.04	0.03
Synechococcus	13.11	0.00	0.14	0.03	0.64	0.80	0.00
Picoeukaryotes	8.74	0.01	0.74	0.11	0.03	0.17	0.74
All	5.25	0.01	0.35	0.27	0.24	0.50	0.82
12.7°N							
Prochlorococcus	3.81	0.03	0.38	0.48	0.42	1.00	0.47
Synechococcus	12.61	0.00	1.00	0.90	0.57	0.00	0.60
Picoeukaryotes	38.51	0.00	0.69	0.02	0.05	0.83	0.00
All	6.79	0.00	0.83	0.99	0.09	0.00	0.02
7.3°S							
Prochlorococcus	3.62	0.03	1.00	0.33	0.02	0.23	0.07
Synechococcus	6.02	0.01	0.01	0.01	0.00	0.11	0.01
Picoeukaryotes	34.39	0.00	0.03	0.53	0.00	0.00	0.26
All	25.48	0.00	0.08	0.34	0.00	0.00	0.75
26.7°S							
Prochlorococcus	21.13	0.00	0.23	0.00	0.01	0.02	0.04
Synechococcus	6.48	0.00	0.15	0.19	0.14	1.00	0.61
Picoeukaryotes	6.68	0.00	1.00	1.00	0.04	0.01	0.03
All	4.04	0.02	0.92	0.65	0.27	0.05	0.10

Supplementary Table 2. Carbon biomass (µg C L^{-1}) of large nanophytoplankton and microphytoplankton (cells > 10 µm in ESD) at t = 96 h for each experiment and treatment. Shown are the mean values for each treatment and in brackets the standard deviation (n = 3). Carbon biomass was calculated from FlowCAM measurements of cell size and abundance and an empirical biovolume to carbon conversion formula. See Methods for details.

	Treatment					
	−3°C	in situ	+3°C	−3°C +Nut	in situ +Nut	+3°C +Nut
28.7°N	0.19 (0.12)	0.23 (0.09)	0.64 (0.15)	0.28 (0.32)	0.54 (0.10)	0.63 (0.28)
12.7°N	0.73 (0.70)	1.16 (0.81)	0.43 (0.17)	1.49 (0.86)	2.07 (0.91)	0.42 (0.12)
7.3°S	0.44 (0.00)	0.65 (0.37)	0.77 (0.28)	0.30 (0.17)	1.02 (0.05)	0.38 (0.11)
26.7°S	0.55 (0.35)	0.57 (0.25)	0.27 (0.12)	0.72 (0.18)	0.40 (0.17)	0.33 (0.06)

Supplementary Table 3. Results of the two-way ANOVA of temperature (Temp.) and nutrient addition (Nut.) effects on final biomass (t = 96 h) of the three picophytoplankton groups and total picophytoplankton biomass (All) in the four experiments.

		28.7°N			12.7°N			7.3°S			26.7°S		
		df	F	*p* value	df	F	*p* value	df	F	*p* value	df	F	*p* value
Prochlorococcus	Temp.	2	0.95	0.42	2	9.53	0.03	2	1.68	0.23	2	15.15	0.00
	Nut.	1	15.25	0.00	1	0.00	0.96	1	12.48	0.00	1	58.85	0.00
	Temp. × Nut.	2	0.61	0.56	2	0.00	1.00	2	1.13	0.35	2	8.24	0.01
Synechococcus	Temp.	2	31.09	0.00	2	14.74	0.00	2	11.67	0.00	2	15.83	0.00
	Nut.	1	3.35	0.09	1	13.67	0.00	1	3.93	0.07	1	0.28	0.61
	Temp. × Nut.	2	0.00	0.99	2	9.96	0.00	2	1.42	0.28	2	0.23	0.80
Picoeukaryotes	Temp.	2	4.24	0.41	2	57.85	0.00	2	33.90	0.00	2	0.25	0.78
	Nut.	1	33.03	0.00	1	42.20	0.00	1	93.54	0.00	1	32.52	0.00
	Temp. × Nut.	2	1.10	0.36	2	17.34	0.00	2	5.31	0.02	2	0.20	0.82
All	Temp.	2	1.45	0.27	2	0.34	0.72	2	28.41	0.00	2	1.70	0.22
	Nut.	1	20.82	0.00	1	28.96	0.00	1	63.09	0.00	1	15.43	0.00
	Temp. × Nut.	2	1.26	0.32	2	2.17	0.16	2	3.74	0.06	2	0.68	0.53

Supplementary Table 4. Analysis of differences in red fluorescence per cell between treatments and the control. The table shows the results of the one-way ANOVA test and the *p*-values for the subsequent Dunnett's post-hoc test to assess the difference in red fluorescence (dependent variable) between each temperature-nutrient treatment (independent variable) and the control. The analysis was carried out with the untransformed cellular red fluorescence data for each picophytoplankton group at t = 96 h on each experiment.

	ANOVA		Post-hoc *p*-value				
	F(5,12)	*p*-value	−3˚C	+3˚C	−3˚C +Nut	in situ +Nut	+3˚C +Nut
28.7°N							
Prochlorococcus	35.77	0.00	1.00	0.13	0.71	0.08	0.00
Synechococcus	6.85	0.00	0.01	0.90	0.02	0.02	0.00
Picoeukaryotes	10.20	0.00	0.41	0.02	0.02	0.01	0.00
12.7°N							
Prochlorococcus	11.75	0.00	0.90	0.14	1.00	0.98	0.00
Synechococcus	23.62	0.00	0.57	0.53	0.56	1.00	0.00
Picoeukaryotes	64.57	0.00	1.00	0.03	0.33	0.44	0.00
7.3°S							
Prochlorococcus	219.95	0.00	0.11	0.00	0.85	0.00	0.00
Synechococcus	10.82	0.00	0.95	0.06	0.11	0.02	0.00
Picoeukaryotes	12.15	0.00	0.43	0.17	0.00	0.00	0.00
26.7°S							
Prochlorococcus	33.67	0.00	0.32	0.19	0.99	0.03	0.00
Synechococcus	8.30	0.00	0.99	1.00	0.99	0.21	0.00
Picoeukaryotes	4.97	0.01	0.73	1.00	0.23	0.00	0.12

Supplementary Table 5. Picophytoplankton red fluorescence relative to the control for each treatment and experiment. Red fluorescence per cell of *Prochlorococcus*, *Synechococcus* and picoeukaryotes in each treatment was divided by the value in the control. Shown is the mean ratio (n = 3) for the three groups combined for each experiment, as well as the mean of the ratios for all locations and its standard deviation (n = 4).

	−3°C	+3°C	−3°C+Nut	in situ+Nut	+3°C+Nut
28.7°N	1.36	1.30	1.43	1.52	2.18
12.7°N	0.75	1.63	1.22	1.17	3.41
7.3°S	1.05	1.26	1.25	1.39	1.79
26.7°S	0.96	0.94	1.06	1.29	1.54
Mean	1.03	1.28	1.24	1.34	2.23
SD	0.18	0.11	0.10	0.06	0.23

Supplementary Table 6. Results of the two-way ANOVA of temperature (Temp.) and nutrient addition (Nut.) effects on cellular chlorophyll fluorescence of the three picophytoplankton groups.

		28.7°N			12.7°N			7.3°S			26.7°S		
		df	F	*p* value	df	F	*p* value	df	F	*p* value	df	F	*p* value
Prochlorococcus	Temp.	2	46.03	0.00	2	22.87	0.00	2	326.19	0.00	2	19.87	0.00
	Nut.	1	53.28	0.00	1	7.53	0.02	1	278.13	0.00	1	78.53	0.00
	Temp. × Nut.	2	16.75	0.00	2	2.74	0.10	2	84.62	0.00	2	25.05	0.00
Synechococcus	Temp.	2	4.91	0.03	2	30.86	0.00	2	12.56	0.00	2	4.87	0.03
	Nut.	1	13.09	0.00	1	33.26	0.00	1	26.84	0.00	1	21.69	0.00
	Temp. × Nut.	2	5.67	0.02	2	11.57	0.00	2	1.07	0.37	2	5.04	0.03
Picoeukaryotes	Temp.	2	10.90	0.00	2	91.38	0.00	2	3.94	0.05	2	0.67	0.53
	Nut.	1	27.05	0.00	1	73.73	0.00	1	51.66	0.00	1	17.50	0.00
	Temp. × Nut.	2	1.08	0.37	2	33.18	0.00	2	0.61	0.56	2	3.00	0.09

Supplementary Table 7. Mean increase (Δ) in macronutrient concentration (μM) after the nutrient amendment at the beginning of each experiment. The standard deviation (n = 2-3), calculated by propagation of uncertainty, is given in brackets.

	$\Delta\ [NO_3^- + NO_2^-]$	$\Delta\ [NH_4^+]$	$\Delta\ [HPO_4^{2-}]$
28.7°N	0.85 (0.04)	0.74 (0.12)	0.16 (0.05)
12.7°N	0.95 (0.34)	0.91 (0.14)	0.14 (0.04)
7.3°S	0.82 (0.24)	1.22 (0.03)	0.15 (0.02)
26.7°S	0.98 (0.24	0.59 (0.07)	0.18 (0.05)
