# Supplementary material for: Phytoplankton responses to changing temperature and nutrient availability are consistent across the tropical and subtropical Atlantic
Source: Commun Biol. 2022 Sep 29;5:1035. doi: 10.1038/s42003-022-03971-z (PMC9522883; doi:10.1038/s42003-022-03971-z)
Supplement: Supplementary file 3 — Description of Additional Supplementary Files [file 42003_2022_3971_MOESM3_ESM.pdf]

Description of Additional Supplementary Files

File name: Supplementary Data 1

Description: Sea surface temperature measured with a CTD probe (SeaBird, SBE, 911plus/917) at the sampling stations during Atlantic Meridional Transect 29.

File name: Supplementary Data 2

Description: Depth profiles of chlorophyll *a* fluorescence at the sampling stations during Atlantic Meridional Transect 29.

File name: Supplementary Data 3

Description: Downwelling, above-water photosynthetically active radiation (PAR, W m^{-2}), measured with a Satlantic HyperSAS system, at the four sampling locations where the experiments were conducted.
