# Supplementary material for: Phytoplankton responses to changing temperature and nutrient availability are consistent across the tropical and subtropical Atlantic
Source: Commun Biol. 2022 Sep 29;5:1035. doi: 10.1038/s42003-022-03971-z (PMC9522883; doi:10.1038/s42003-022-03971-z)
Supplement: Supplementary file 7 — Reporting Summary [file 42003_2022_3971_MOESM7_ESM.pdf]

nature portfolio

Corresponding author(s): Emilio Marañón

Last updated by author(s): Aug 26, 2022

Reporting Summary

Nature Portfolio wishes to improve the reproducibility of the work that we publish. This form provides structure for consistency and transparency in reporting. For further information on Nature Portfolio policies, see our Editorial Policies and the Editorial Policy Checklist.

Statistics

For all statistical analyses, confirm that the following items are present in the figure legend, table legend, main text, or Methods section.

n/a	Confirmed	
☐	☒	The exact sample size (*n*) for each experimental group/condition, given as a discrete number and unit of measurement
☐	☒	A statement on whether measurements were taken from distinct samples or whether the same sample was measured repeatedly
☐	☒	The statistical test(s) used AND whether they are one- or two-sided<br>*Only common tests should be described solely by name; describe more complex techniques in the Methods section.*
☒	☐	A description of all covariates tested
☐	☒	A description of any assumptions or corrections, such as tests of normality and adjustment for multiple comparisons
☐	☒	A full description of the statistical parameters including central tendency (e.g. means) or other basic estimates (e.g. regression coefficient) AND variation (e.g. standard deviation) or associated estimates of uncertainty (e.g. confidence intervals)
☐	☒	For null hypothesis testing, the test statistic (e.g. *F*, *t*, *r*) with confidence intervals, effect sizes, degrees of freedom and *P* value noted<br>*Give P values as exact values whenever suitable.*
☒	☐	For Bayesian analysis, information on the choice of priors and Markov chain Monte Carlo settings
☒	☐	For hierarchical and complex designs, identification of the appropriate level for tests and full reporting of outcomes
☒	☐	Estimates of effect sizes (e.g. Cohen's *d*, Pearson's *r*), indicating how they were calculated

Our web collection on statistics for biologists contains articles on many of the points above.

Software and code

Policy information about availability of computer code

Data collection	VisualSpreadsheet© Particle Analysis Sofware v4, CellQuest software v3 (Becton Dickinson, Oxford)
Data analysis	SPSS v.22 (IBM, Armonk, NY, USA) and R studio v 4.0.3 (RStudio, Boston, Massachusetts, USA)

For manuscripts utilizing custom algorithms or software that are central to the research but not yet described in published literature, software must be made available to editors and reviewers. We strongly encourage code deposition in a community repository (e.g. GitHub). See the Nature Portfolio guidelines for submitting code & software for further information.

Data

Policy information about availability of data

All manuscripts must include a data availability statement. This statement should provide the following information, where applicable:

- Accession codes, unique identifiers, or web links for publicly available datasets
- A description of any restrictions on data availability
- For clinical datasets or third party data, please ensure that the statement adheres to our policy

All data have been submitted for archiving at the British Oceanographic Data Centre (BODC) https://www.bodc.ac.uk/. The doi to access the data is given in the manuscript.

Human research participants

Policy information about studies involving human research participants and Sex and Gender in Research.

Reporting on sex and gender	*Use the terms sex (biological attribute) and gender (shaped by social and cultural circumstances) carefully in order to avoid confusing both terms. Indicate if findings apply to only one sex or gender; describe whether sex and gender were considered in study design whether sex and/or gender was determined based on self-reporting or assigned and methods used. Provide in the source data disaggregated sex and gender data where this information has been collected, and consent has been obtained for sharing of individual-level data; provide overall numbers in this Reporting Summary. Please state if this information has not been collected. Report sex- and gender-based analyses where performed, justify reasons for lack of sex- and gender-based analysis.*
Population characteristics	*Describe the covariate-relevant population characteristics of the human research participants (e.g. age, genotypic information, past and current diagnosis and treatment categories). If you filled out the behavioural & social sciences study design questions and have nothing to add here, write "See above."*
Recruitment	*Describe how participants were recruited. Outline any potential self-selection bias or other biases that may be present and how these are likely to impact results.*
Ethics oversight	*Identify the organization(s) that approved the study protocol.*

Note that full information on the approval of the study protocol must also be provided in the manuscript.

Field-specific reporting

Please select the one below that is the best fit for your research. If you are not sure, read the appropriate sections before making your selection.

☐ Life sciences ☐ Behavioural & social sciences ☒ Ecological, evolutionary & environmental sciences

For a reference copy of the document with all sections, see nature.com/documents/nr-reporting-summary-flat.pdf

Ecological, evolutionary & environmental sciences study design

All studies must disclose on these points even when the disclosure is negative.

Study description	Samples of microbial plankton were enclosed in 1-L bottles and subjected to all combinations of three temperatures (in situ, 3°C cooling, 3°C warming) and two nutrient availability regimes (unamended and enriched with N and P), then monitored daily during 96h.
Research sample	Each experimental sample consisted of a 1-L bottle containing the microbial plankton (unfiltered seawater)
Sampling strategy	Each day, individual samples were obtained for the determination of active Chl a fluorescence parameters and Prochlorococcus, Synechococcus and picoeukaryotic abundances. In addition, at the beginning and end of the incubations, samples were taken to determine the abundance of microphytoplankton and the concentration of Chl a, while nutrient concentration was determine only at the beginning of the experiments.
Data collection	All samples were collected by Cristina Fernández-González who also measured extracted Chl a concentration. Glen Tarran determined picophytoplankton abundances by flow cytometry, Nina Schuback measured active Chl a fluorescence parameters by using a FRRF, Javier Arístegui determined microphytoplankton abundances by FlowCam and Malcolm Woodward measured nutrient concentrations.
Timing and spatial scale	The location and starting date for each experiment were as follows: Expt 1: 48.7°N, 33°W, Oct 26, 2019; Expt 2: 12.7°N, 28.5 °W, Nov 1, 2019; Expt. 3: 7.3°S, 25°W, Nov 7, 2019; Expt 4: 26.7°S, 25.8°W, Nov 12, 2019. All experiments lasted for 96 h.
Data exclusions	No data were excluded form the analysis.
Reproducibility	The same experimental design was repeated on 4 occasions during the oceanographic cruise, at the four geographical locations given above (see also Table 1 of manuscript).
Randomization	Experimental units (1-L bottles) were assigned randomly to each temperature x nutrient treatment
Blinding	Blinding is not possible in this experimental design, since different bottles were incubated in different tanks at different temperatures. However, processing of all samples was conducted in the same way, irrespective of treatment.

Did the study involve field work? ☒ Yes ☐ No

Field work, collection and transport

Field conditions	Multiple in situ conditions at each sampling location, including sea surface temperature, are given in Table 1 of the manuscript.
Location	The location and starting date for each experiment were as follows: Expt 1: 48.7°N, 33°W, Oct 26, 2019; Expt 2: 12.7°N, 28.5 °W, Nov 1, 2019; Expt. 3: 7.3°S, 25°W, Nov 7, 2019; Expt 4: 26.7°S, 25.8°W, Nov 12, 2019.
Access & import/export	*Describe the efforts you have made to access habitats and to collect and import/export your samples in a responsible manner and in compliance with local, national and international laws, noting any permits that were obtained (give the name of the issuing authority, the date of issue, and any identifying information).*
Disturbance	*Describe any disturbance caused by the study and how it was minimized.*

Reporting for specific materials, systems and methods

We require information from authors about some types of materials, experimental systems and methods used in many studies. Here, indicate whether each material, system or method listed is relevant to your study. If you are not sure if a list item applies to your research, read the appropriate section before selecting a response.

Materials & experimental systems

n/a	Involved in the study
☒	☐ Antibodies
☒	☐ Eukaryotic cell lines
☒	☐ Palaeontology and archaeology
☒	☐ Animals and other organisms
☒	☐ Clinical data
☒	☐ Dual use research of concern

Methods

n/a	Involved in the study
☒	☐ ChIP-seq
☐	☒ Flow cytometry
☒	☐ MRI-based neuroimaging

Flow Cytometry

Plots

Confirm that:

☐ The axis labels state the marker and fluorochrome used (e.g. CD4-FITC).

☐ The axis scales are clearly visible. Include numbers along axes only for bottom left plot of group (a 'group' is an analysis of identical markers).

☐ All plots are contour plots with outliers or pseudocolor plots.

☒ A numerical value for number of cells or percentage (with statistics) is provided.

Methodology

Sample preparation	Every 24 h we took 6-mL samples from the incubation bottles (unfiltered phytoplankton natural assemblages) in Falcon tubes that were transported immediately into the lab and kept in dark at 4°C until analysis by analytical flow cytometry. Samples were analysed live.
Instrument	BD FACSort
Software	CellQuest version 3
Cell population abundance	Not applicable
Gating strategy	All analyses were primarily thresholded using chlorophyll (CHL) fluorescence. This enabled discrimination of algae from other particles. For picocyanobacteria (Synechococcus sp and Prochlorococcus sp) an inital plot of phycoerythrin (PE) fluorescence vs. CHL fluorescence was used. A region was drawn around the Synechococcus (region R1). This region enabled enumeration of the Synechococcus. A second plot of side scatter vs. CHL fluorescence was used in which the Synechococcus data were removed using region R1. A second region (R2) was drawn around the Prochlorococcus to enumerate them. For picoeukaryote algae an initial plot of PE fluorescence vs. CHL fluorescence was used. A region (R3) was drawn around all events with significant PE fluorescence. A second plot of side scatter vs. CHL fluorescence was then used in which the events in region R3 were removed. A region (R4) was then drawn around the picoeukaryote cluster to enumerate them.

☐ Tick this box to confirm that a figure exemplifying the gating strategy is provided in the Supplementary Information.
